# Susceptibility based magnetic resonance imaging for development of neuroimaging biomarkers

**DOI:** 10.1002/alz.087694

**Published:** 2025-01-09

**Authors:** Lauren F O'Donnell, Suleiman Khan, Hannah Goldman, Sean Murray, Jakub Szabo, Muhammad T Soliman, Michael Llanos, Olivia Cho, Charles Kingsley, Jody Swain, Stanton B Gray, William D Hopkins, Thomas Wisniewski, Jelle Veraart, Henrieta Scholtzova, Youssef Zaim‐Wadghiri

**Affiliations:** ^1^ Bernard and Irene Schwartz Center for Biomedical Imaging, New York University Grossman School of Medicine, New York, NY USA; ^2^ Center for Advanced Imaging Innovation and Research (CAI2R), New York University Grossman School of Medicine, New York, NY USA; ^3^ NYU Grossman School of Medicine, New York, NY USA; ^4^ New York University Grossman School of Medicine, New York, NY USA; ^5^ Small Animal Imaging Facility, UT MD Anderson Cancer Center, Houston, TX USA; ^6^ UT MD Anderson Cancer Center, Michale E. Keeling Center for Comparative Medicine and Research, Bastrop, TX USA; ^7^ Alzheimer's Disease Research Center, New York University Langone Health, New York, NY USA

## Abstract

**Background:**

Amyloid related imaging abnormalities (ARIA), a group of neuropathological features seen in anti‐amyloid immunotherapy patients, arises partly from CAA (Aβ buildup in blood vessels). Squirrel monkeys (SQMs), developing prominent age‐related CAA exceeding brain Aβ, offer a unique NHP model for ARIA study. Evaluating edema‐related neurobiological defects (ARIA‐E) involves preferential use of T_2_‐weighted (T_2_‐w) and flow‐attenuated inversion recovery (FLAIR) MRI while T_2_*‐weighted (T_2_*‐w) MRI is better suited for investigating iron‐related pathology like microbleeds, hemorrhaging, and iron‐homing in plaques. Further insights can be gained by mapping tissue magnetic susceptibility, which boosts sensitivity and specificity. Susceptibility‐weighted imaging (SWI) achieves this by combining magnitude and phase images to enhance contrast from iron containing structures. Notably, quantitative susceptibility mapping (QSM) goes a step further by utilizing phase information with field inversion computation to generate quantitative magnetic susceptibility values in the imaged tissue. This ability to differentiate between paramagnetic (iron) and diamagnetic (calcification) signal contributions makes QSM valuable for potentially quantifying iron content more accurately. Here we present the sensitivity of conventional T_2_* weighted and quantitative R_2_* (QR2*) imaging to SWI and QSM analysis in this SQM model.

**Method:**

In Vivo MRI scans were acquired on a cohort of geriatric SQMs using a multi‐sequence protocol: a multigradient echo (MGE) sequence for susceptibility mapping, a 2D T_2_‐w Turbo‐spin‐echo for edema assessment, and 2D FLAIR for improved white matter and edema visualization. Susceptibility maps were generated from the MGE data using both SWI reconstruction (PV6.0.1, Bruker) and the QSM processing in STI Suite for Matlab by Liu et al. (2013).

**Result:**

Expanding on QR2*'s success in aging SQM iron pathology, we compared QSM and SWI to it and T2*‐w, uncovering latent pathologies. QSM revealed iron in hyper‐intense regions with heightened SWI contrast, and conversely, identified unspecific hypo‐intensities, likely calcification, in some subjects. Immunohistochemistry then characterized these MRI anomalies.

**Conclusion:**

Susceptibility imaging leverages tissue's magnetic properties to reveal subtle iron deposits, microbleeds, and calcifications that often escape detection. This unveils invaluable insights into iron‐related pathologies in the brain, invisible to conventional MRI's proton‐based techniques.

